# Notes and News

**Published:** 1905-11-04

**Authors:** 


### Notes and News.

Princess Christian opened a bazaar in aid of the Darlington Hospital on Wednesday.

Through the will of the late Mr. Maxwell Armstrong, of Dublin, the Adelaide Hospital, Dublin, will share, with several other charities, in the residue of his estate.

It is surprising how successful bazaars in aid of local charities are in the country. The Kelso Hospital Bazaar has recently produced £2,500, and the interest it has created is still benefiting the hospital.

There is great activity throughout the kingdom in the establishment of sanatoria for consumption. At Leeds there is a proposal, originated by a medical man, to utilise the Winter Street Hospital for consumptives. In Aberdeen Lady Lumsden has offered £1,000, and the Clerk Trustees £7,000 for a sanatorium for the city.

Messrs. S. Van Royen and Barghoorn, of Water Lane, have signified their intention to import only butter from Holland bearing the Government stamp. They have just been summoned by the Customs for selling butter adulterated with foreign fat. A nominal fine was inflicted, as the firm appeared to have taken precautions to insure the receipt of pure butter.

The Mount Vernon Hospital for Consumption and Diseases of the Chest has received a donation of £1,000 from the Misses Isabel and Emma Goldsmid in memory of the late Miss Flora Goldsmid, in response to their appeal for £100,000 for the purpose of opening the 110 empty beds in the hospital. We can imagine nothing more convincing than a visit to the Northwood branch of the hospital. The patients of the occupied wards are so obviously improved by their sojourn at Northwood that it seems a terrible pity that a larger number cannot be maintained and received into the empty rooms of the upper floor of the hospital, to benefit from the excellent air and beautiful surroundings.

Dr. H. Leigh Canney will lecture on "The Toleration of Enteric Fever by the Army," at the Royal Institute, on November 8.

The Royal Commission on the Care of the Feeble-minded continues its sittings, and a very large number of witnesses have already been heard.

The annual dinner of the Poplar Hospital will be held on November 13 at the Holborn Restaurant. Mr. Edgar Speyer will preside.

The annual dinner of the West London Hospital will be held on December 14 at the Trocadero Restaurant. The Duke of Abercorn will preside.

The late Miss Katherine Hall, of Bordighera, Italy, has bequeathed £2,500 each to the Cancer Hospital, University Hospital, and the Hospital for Sick Children, Great Ormond Street.

The National Committee for the Establishment of Sanatoria for Workers has received handsome contributions from Lord and Lady Cadogan, Lord Iveagh, Lord and Lady Rothschild, and others.

Dr. G. Murray will deliver the Bradshaw Lecture before the College of Physicians on November 7. The subject is "Exophthalmic Goitre and its Treatment."

The Chairman of the Passmore Edwards Hospital at Willesden is appealing for aid to purchase the site on which the institution stands, and adjoining land which is available, for the extension which will sooner or later be necessary. The population of Willesden is now 140,000, and is rapidly increasing. The hospital now contains twenty-four beds.

A milk-vendor in Long Lane has been fined £10 for selling milk with the addition of 7 per cent. of water. The presiding magistrate imposed this heavy fine as the opinion is held that the fines demanded do not, as a rule, prevent adulteration. It has been recently demonstrated that all milk that comes into the City is above the standard fixed by the Board of Agriculture.

When we attend a demonstration of Ju-Jitsu it appears so simple and playful that it is difficult to realise what a practical force its acquisition imparts. Now it is reported from Paris that a professional boxer and a professor of Ju-Jitsu had an encounter, which resulted in the defeat of the boxer in ten seconds. Ju-Jitsu is a healthy exercise, but, as it has a protective use, it serves two purposes.

St. Mary's Hospital, the Manchester and Salford Lying-in Hospital and Dispensary, the Southern Hospital for Women and Children, and the Manchester Maternity Hospital have now arranged to amalgamate under the name of the St. Mary's Hospitals. A new hospital will be built in Oxford Street, as part of the combination; accommodation will be provided for six resident medical students, twelve resident midwifery and monthly nursing pupils, in Whitworth Street West.

